# Bovine-associated CNS species resist phagocytosis differently

**DOI:** 10.1186/1746-6148-9-227

**Published:** 2013-11-11

**Authors:** Silja Åvall-Jääskeläinen, Joanna Koort, Heli Simojoki, Suvi Taponen

**Affiliations:** 1 Department of Veterinary Biosciences, Division of Microbiology and Epidemiology, University of Helsinki, P.O. Box 66, FIN-00014, Helsinki, Finland; 2 Department of Production Animal Medicine, Faculty of Veterinary Medicine, University of Helsinki, P.O. Box 57, FIN-00014, Helsinki, Finland

## Abstract

**Background:**

Coagulase-negative staphylococci (CNS) cause usually subclinical or mild clinical bovine mastitis, which often remains persistent. Symptoms are usually mild, mostly only comprising slight changes in the appearance of milk and possibly slight swelling. However, clinical mastitis with severe signs has also been reported. The reasons for the differences in clinical expression are largely unknown. Macrophages play an important role in the innate immunity of the udder. This study examined phagocytosis and killing by mouse macrophage cells of three CNS species: *Staphylococcus chromogenes* (15 isolates), *Staphylococcus agnetis* (6 isolates) and *Staphylococcus simulans* (15 isolates). *Staphylococcus aureus* (7 isolates) was also included as a control.

**Results:**

All the studied CNS species were phagocytosed by macrophages, but *S. simulans* resisted phagocytosis more effectively than the other CNS species. Only *S. chromogenes* was substantially killed by macrophages. Significant variations between isolates were seen in both phagocytosis and killing by macrophages and were more common in the killing assays. Significant differences between single CNS species and *S. aureus* were observed in both assays.

**Conclusion:**

This study demonstrated that differences in the phagocytosis and killing of mastitis-causing staphylococci by macrophages exist at both the species and isolate level.

## Background

Mastitis is a major disease of dairy cows that is most often caused by bacteria such as staphylococci, streptococci and coliforms. *Staphylococcus aureus* and coagulase-negative staphylococci (CNS) are isolated in about half of mastitic milk samples in Finland [1] and in many other countries [2-4]. The manifestation of staphylococcal mastitis varies considerably. It is well-known that *S. aureus* is able to cause severe clinical signs, but generally it causes persistent subclinical mastitis. CNS generally cause subclinical or mild clinical mastitis [5], although clinical CNS mastitis with severe signs has also been reported [4,6]. Self-clearance of CNS mastitis soon after parturition is commonly expected [7,8], but CNS have been shown to frequently persist in the udder throughout lactation, causing an elevated somatic cell count (SCC) [9-11].

Phagocytic leucocytes form the primary cellular defence of the udder [12]. During lactation, normal milk from a healthy bovine udder contains a small number of leucocytes, usually <50 000 cells/ml [9,12]. These cells, which are first in line to meet possible intruders, are mainly macrophages [12]. The role of these macrophages in either the prevention or induction of inflammation is crucial. Among several other functions, they recognize microorganisms, alert the immune system and initiate an inflammatory reaction (reviewed, for example, in [13]). Only after the initiation of inflammation are polymorphonuclear neutrophils (PMNs) enrolled, which move from the blood into the milk, raising the SCC in the milk to hundreds of thousands or millions cells/ml. *S. aureus* is well known for its ability to evade phagocytosis [14,15] and persist in the udder [11]. For example, *S. aureus* expresses several antiopsonic cell-surface components, including a polysaccharide capsule and surface protein A, which enable the bacteria to efficiently resist phagocytosis. This species also has several mechanisms allowing it to survive within phagocytic cells, including enzymes that neutralize free radicals [14]. CNS are also known to persist in the udder [9-11], which implies that, like *S. aureus*, they have developed means to resist phagocytosis. However, the virulence factors related to the putative ability of CNS from bovine mastitis to resist phagocytosis are still poorly characterized.

Our aim was to investigate the resistance to phagocytosis of the bovine-associated CNS species *S. chromogenes*, *S. simulans* and *S. agnetis*, and to examine possible differences in resistance to phagocytosis and killing between these staphylococcal species and isolates. *S. chromogenes* and *S. simulans* were selected for study due to their isolation frequencies, and *S. agnetis* to gain information on this recently described species. *S. aureus* was included as a control. Macrophages were used to represent the primary stage of cell-mediated innate immunity, and a commercial mouse macrophage cell line was selected to achieve stable growth characteristics. Here, we report differences between species and isolates in the phagocytosis and killing of mastitis-causing staphylococci by macrophages.

## Methods

### Bacterial isolates and growth conditions

The 43 *Staphylococcus* isolates used in this study are listed in Table 1. The isolates were selected on the basis of their clinical properties, and included isolates from clinical or subclinical mastitis as well as from persistent or transient intramammary infection. Almost all isolates originated from different dairy herds, and they were likely to be genetically different. As a reference, isolates from extramammary sites and type strains of *Staphylococcus* were also included in the study. Staphylococci were routinely grown in Mueller Hinton (LabM, Bury, UK) at +37°C. The strains and isolates were maintained at -80°C in Mueller Hinton broth containing 8.7% (vol/vol) glycerol.

**Table 1:** **The staphylococcal isolates used in the phagocytosis assay**^**1**^

	**Source of isolates**						
**Species**	**Clinical mastitis**	**Subclinical mastitis**	**Persistent subclinical mastitis**	**Transient subclinical mastitis**	**Extra-mammary sites**	**Type strain**	**Total number**
*S. chromogenes*	4	4	2	2	2	1	15
*S. simulans*	4	4	2	2	2	1	15
*S. agnetis*	3*	3				(1)*	6
*S. aureus*	2	4				1	7
Total number	13	15	4	4	4	4	

^1^The isolates from clinical or subclinical mastitis, except for two *S. aureus* isolates from subclinical mastitis, originate from the data of [16]. Two *S. aureus* isolates from subclinical mastitis originate from the data of Taponen et al. [17]. The isolates from persistent or transient bovine mastitis originate from the study of Taponen et al. [9] and the isolates from extramammary sites (skin of the udder, teats, perineum, and the milker’s hand) originate from the study of Taponen et al. [18].

*One of the *S. agnetis* isolates from clinical mastitis is the *S. agnetis* type strain DSM 23656^T^.

### Cell culture

The murine macrophage cell line J774, derived from BALB/cN reticulum cell sarcoma (ATCC TIB-67; American Type Culture Collection, Rockville, Md., USA), was maintained at +37°C with 5% CO_2_ in growth medium (GM) containing Dulbecco Modified Eagle medium (DMEM High Glucose, GlutaMAX™; Gibco, Grand Island, NY, USA) supplemented with 10% heat-inactivated foetal bovine serum (Gibco). Cells were maintained as monolayers in 75 cm^2^ flasks and passaged two or three times weekly by scraping and diluting the cells into fresh DMEM with 5% foetal bovine serum.

### *In vitro* phagocytosis and killing assay

The staphylococci to be used in the phagocytosis and killing assays were grown overnight in Mueller Hinton broth. The OD_600nm_ of the cultures was determined, and the cells were collected by centrifugation, washed once with phosphate-buffered saline (PBS) and resuspended in GM to an approximate concentration of 6 × 10^6^ colony forming units (CFU)/ml. Cells were divided into aliquots and stored at -80 C until further use. Before the infection assays, the viability of the frozen *Staphylococcus* cells was determined by thawing them at +37°C in a water bath for 30 min, followed by suitable dilution in buffered peptone water (LabM, Bury, UK) and plating on Mueller Hinton agar. The number of viable *Staphylococcus* cells was determined by counting the CFU after overnight incubation (20–23 h).

The *in vitro* phagocytosis and killing assays were performed essentially as described previously [19], with some modifications. Murine J774 cells were seeded into 24-well microtiter plates (Greiner Bio-One, Frickenhausen, Germany) at a density of 1 × 10^5^ cells per well (volume 800 μl). After 24 h, these cells were infected with 1 × 10^5^ of *Staphylococcus* cells (in 50 μl volume) derived from frozen aliquots. Before addition to plates, the bacteria were thawed for 30 min in a water bath at 37°C and diluted in fresh GM. The plates were centrifuged shortly after the addition of bacteria (5 min 180 g RT). Thereafter, the plates were incubated for 1 h at +37°C with 5% CO_2_.

After incubation, the treatment of plates differed between phagocytosis and killing assays. In the killing assay, the J774 cells were lysed by incubating them in GM with 0.1% saponin (vol/vol; Merck kGaA, Darmstadt, Germany) for 15 min at +37°C with 5% CO_2_. Thereafter, the lysed samples were removed from wells by vigorous pipetting, the wells were washed with PBS and the recovered buffer was combined with the lysate previously recovered from the wells. Aliquots from these samples were plated as described in the viability determination of frozen *Staphylococcus* cells to count the total number of surviving bacteria in the samples. In the phagocytosis assay, after infection, the extracellular bacteria were recovered by carefully removing the GM from the wells. Thereafter, the wells were washed with PBS by cautious pipetting and the recovered buffer was combined with the GM previously recovered from the wells. To count the extracellular bacteria in these samples, plating was carried out as in the killing assay.

As a reference for both assays, staphylococci were incubated simultaneously in plates without J774 cells, and the wells in these plates were treated as in the phagocytosis assay. The numbers of killed staphylococci were calculated by subtracting the counts of surviving staphylococci from the counts of staphylococci in reference samples. The numbers of phagocytosed staphylococci were calculated by subtracting the counts of extracellular staphylococci from the counts of staphylococci in reference samples. The quantities of killed and phagocytosed staphylococci are presented as percentages of reference sample values. Each value represents the mean of three independent experiments, with two replicates for each sample in a single experiment.

### Statistical analysis

Mean values for the phagocytosis and killing of each of the studied *Staphylococcus* species and isolates by murine macrophage cell line J774 were calculated. All results are expressed as arithmetic means ± standard deviation. The studied isolates were grouped on the basis of variables related to the nature of infection (clinical/subclinical) in order to determine whether this isolate attribute has an effect on the phagocytosis or killing of staphylococci by murine J774 macrophages.

The possible effects of staphylococcal species, staphylococcal isolate and the above-mentioned isolate attributes on the phagocytosis and killing of staphylococci by the murine macrophage cell line J774 were examined. The arithmetic means of the three repeated, independent experiments for each isolate were used in the statistical analyses in order to avoid the clustering effect. The independent-samples Kruskal-Wallis test was used to study the possible effects of these factors on phagocytosis, for which the data were not normally distributed. The data for bacterial killing were normally distributed and ANOVA was used to examine the possible effects of these factors on killing. Test results were considered statistically significant if the calculated *p* values were < 0.05.

## Results

### Phagocytosis of staphylococci by the murine macrophage cell line J774

**Figure 1:**
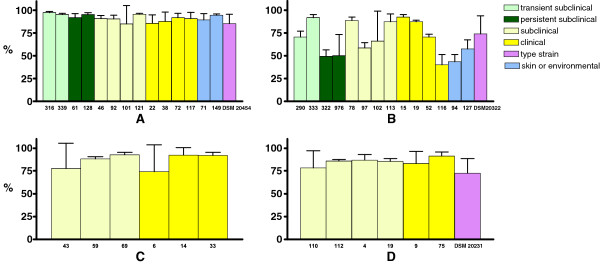
**The mean phagocytosis percentages (±SD) of S*. chromogenes *(A)*, S. simulans *(B), *S. agnetis *(C) and *S. aureus *(D) isolates by murine J774 macrophages.** Bars indicate the average result ± standard deviation of three separate assays, with two replicates of each sample in a single experiment. The origins of the isolates included are marked with different bar colours.

Staphylococci were not resistant to phagocytosis by murine J774 macrophages, since phagocytosis occurred for all the isolates of the four studied *Staphylococcus* species during the one-hour incubation with these cells (Figure 1). Among all the studied staphylococcal isolates, the mean percentages of phagocytosis varied from ~40% (*S. simulans* isolate 116) to ~ 97% (*S. chromogenes* isolate 316). The standard deviation of the mean phagocytosis rates varied from 1.3% (*S. chromogenes* isolate 316) to 32.8% (*S. simulans* isolate 102).

**Figure 2:**
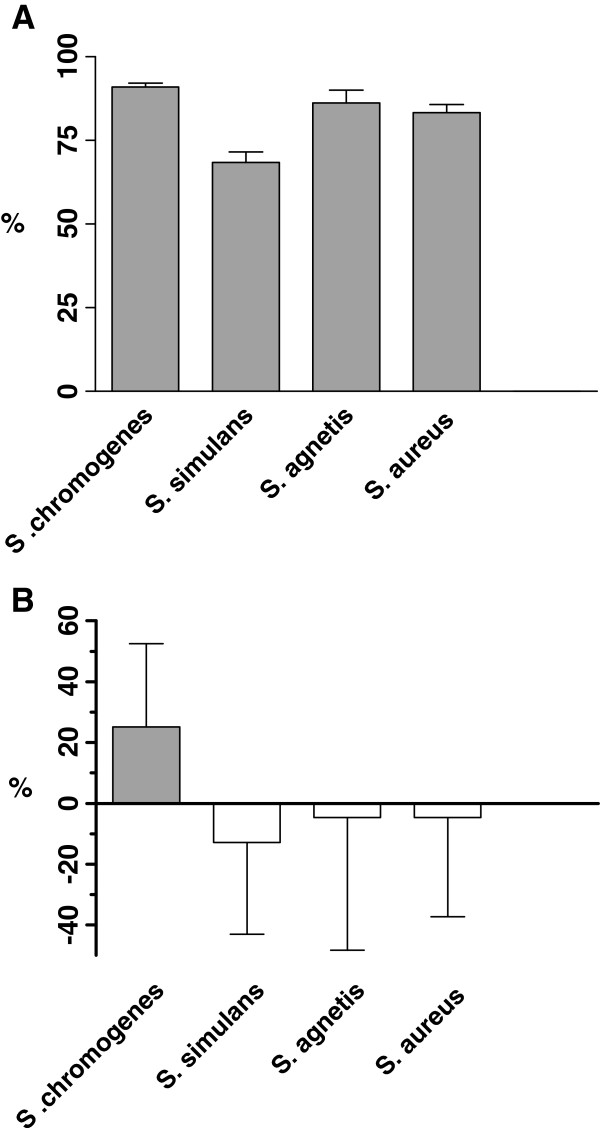
**The mean phagocytosis (A) and killing (B) percentages (±SD) of S*. chromogenes, S. simulans*, *S. agnetis *and *S. aureus *by murine J774 macrophages.***S. simulans* was less phagocytosed than *S. agnetis* (*p* = 0.014) and *S. chromogenes* (*p* < 0.001). *S. aureus* was less phagocytosed than *S. chromogenes* (*p* = 0.020). *S. chromogenes* differed from other species and was killed more efficiently (*p* < 0.05). Negative values for killing are due to the growth of staphylococci during saponin treatment.

The mean percentage of phagocytosis for *S. simulans* was 68.4% ± 21.36%, whereas for the other studied species it varied between 83.3% and 91.0% (Figure 2A). For all the studied *Staphylococcus* species, the median percentage of phagocytosis did not significantly differ from the mean values (results not shown). *S. simulans* was significantly less phagocytosed than *S. agnetis* (*p* = 0.014) and *S. chromogenes* (*p* < 0.001). In contrast, no significant difference in phagocytosis was observed between *S. simulans* and *S. aureus* (*p* = 0.185), while *S. aureus* was significantly less phagocytosed than *S. chromogenes* (*p* = 0.020). No differences in phagocytosis were recorded between isolates derived from either subclinical or clinical infections (*p* = 0.544).

Large and statistically significant variation was recorded in the phagocytosis of different *S. simulans* isolates which varied from 39.9% ± 11.4% to 91.5% ± 3.6%. Such variation between isolates was not observed for the other *Staphylococcus* species.

### Killing of staphylococci by murine macrophage cell line J774

There was considerable variation between bacterial species in the killing of staphylococci by murine J774 macrophages during the one-hour incubation period (Figure 2B). *S. chromogenes* was more efficiently killed by murine J774 macrophages than any of the other *Staphylococcus* species studied. The *p-*values of statistical differences between *S. chromogenes* and *S. simulans, S. agnetis* or *S. aureus* were <0.001, 0.048 and 0.016, respectively. The mean percentage of killing for *S. chromogenes* was 25.10% ± 27.41%, but for the other *Staphylococcus* species the values were negative (Figure 2B). This was most likely due to the multiplication of bacterial cells in the GM during saponin treatment (Åvall-Jääskeläinen et al., unpublished observations).

**Figure 3:**
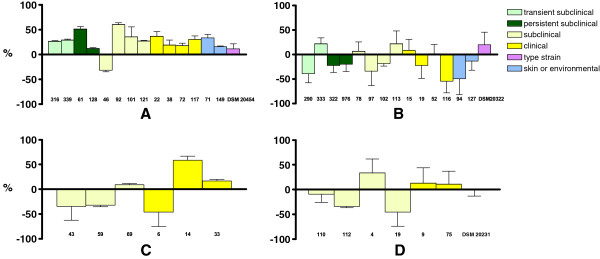
**The mean killing percentages (±SD) of *S. chromogenes *(A)*, S. simulans *(B), *S. agnetis *(C) and *S. aureus *(D) isolates by murine J774 macrophages.** Bars indicate the average result ± standard deviation of three separate assays, with two replicates of each sample in a single experiment. The origins of the strains included are marked with different bar colours.

Substantial differences were detected in the killing of different isolates within a single species for all the studied CNS species. Some of the studied *Staphylococcus* isolates were not killed at all, whereas others were efficiently killed. The maximum mean percentage killing of a single isolate was 60.1% (*S. chromogenes* isolate 92) (Figure 3). No difference was recorded in the killing of isolates derived from either subclinical or clinical infections (*p* = 0.363).

## Discussion

This study examined the ability of macrophages to phagocytose and kill bovine mastitis-associated strains of *S. aureus* and the CNS species *S. simulans*, *S. chromogenes* and *S. agnetis*. This is related to the capability of the bacteria to initiate infection, as resistance to macrophages is crucial, especially in the first stages of infection. As references, extramammary isolates and type strains of these staphylococcal species were also included. All the studied species were phagocytosed, although differences were recorded in the efficacy of phagocytosis. *S. simulans* resisted phagocytosis more than *S. chromogenes* and *S. agnetis.* Inside the macrophages, *S. chromogenes* was more efficiently killed than the other *Staphylococcus* species.

Although the phagocytosis of bovine mastitis-associated *S. aureus* by PMNs [20-22] and macrophages [23,24] has been extensively studied, the phagocytosis of CNS has earlier received little if any research attention. For this study, we selected CNS species that are not only relevant and frequent, but also differ from each other as a cause of bovine mastitis. Thus, *S. chromogenes* and *S. simulans*, species belonging to the few most frequently isolated CNS species in the Nordic countries [4], were chosen. *S. chromogenes* is suspected to belong to the bovine skin microbiota [18,25], and is also the most commonly isolated CNS species in bovine milk, especially in heifers around calving [26] and in first lactation [16]. *S. simulans*, also commonly isolated in mastitic milk samples, especially in Nordic countries [4,16], has been reported to have a tendency to cause a stronger inflammatory reaction than other CNS species [16]. *S. agnetis*, a recently described bovine-associated coagulase-variable species [27], was also included as a new species for which information is still limited. *S. agnetis,* described by our research group, is frequently isolated from mastitic milk samples, but does not belong to the few most commonly isolated CNS species (Taponen, unpublished results). It is closely related to and was earlier classified as *S. hyicus*, which has been reported to cause stronger inflammatory reactions than many other CNS species [28]. Few studies have examined the phagocytosis of *S. simulans*[29-31], and the present study is, to our knowledge, the first examining the phagocytosis of *S. chromogenes* and *S. agnetis*.

In this study we used murine macrophages rather than neutrophils, as in the udder they are the first phagocytes of innate immunity and thus have an important role in the early immune response [13]. To ensure repeatability, we used a commercially available cell line rather than freshly isolated cells. To our knowledge, no commercial bovine macrophage cell lines are available. However, the murine macrophage cell line J774 has stable growth characteristics and has been used in numerous bacterial phagocytosis studies [19,32,33]. In terms of the innate recognition of bacteria, the differences between murine and bovine macrophages are quite small. For example, the selection of toll-like receptors (TLRs), known to be the most important pattern-recognition receptor family, is similar in macrophages of both species, as mouse macrophages possess all the known cell surface TLRs of cattle macrophages. Murine macrophages can thus be used to evaluate trends and differences in phagocytosis between the different bovine-associated bacterial isolates, keeping in mind that the values for phagocytosis obtained this way are not necessarily exactly the same as would be obtained by using bovine macrophages. When phagocytosis and killing were compared between the CNS species, some significant differences were seen. *S. simulans* resisted phagocytosis more effectively than the other CNS species studied, and if phagocytosed, also resisted killing by phagocytes as effectively as *S. agnetis* or even *S. aureus. S. chromogenes,* by comparison, was phagocytosed more easily than *S. simulans*, and also more efficiently killed by phagocytes than any other studied species. Interestingly, these two species also appear to behave differently as udder pathogens. *S. simulans* is commonly isolated in milk samples originating from mastitic udders [4,16,28,34]. In two field studies, a tendency has been observed for *S. simulans* to cause slightly more clinical signs than other CNS species [16,34], and in an experimentally induced bovine mastitis study it caused a slightly stronger inflammatory response when compared to *S. epidermidis*[35]. On the other hand, *S. chromogenes* is known to be commonly isolated in bovine mastitis milk samples, especially from first lactation cows [11,16,26,36], and to colonize the teat and udder skin of heifers before and around calving [25,37]. Even though these species are both usually classified as opportunists, molecular data indicate that *S. simulans* may be specifically associated with mastitis, whereas *S. chromogenes* could be regarded as a species belonging to the bovine skin microbiota [18,37]. This difference is consistent with the observation that *S. chromogenes* in this study was more easily phagocytosed and killed than *S. simulans.* However, despite their differences, both *S. simulans* and *S. chromogenes* commonly persist in the udder, even for an entire lactation, causing an elevated milk SCC [9,11]. In experimentally induced mastitis [35], six of eight *S. simulans* mastitis infections remained persistent.

This study was the first to examine the phagocytosis of *S. agnetis*, which is a newly described coagulase-variable staphylococcal species isolated from subclinical and mild clinical bovine mastitis [27]. In this study it is regarded as coagulase-negative, as only few strains are coagulase-positive and production of coagulase is slow compared to that of *S. aureus*[27]*.* Most of the known *S. agnetis* isolates have previously been misidentified as *S. hyicus* by phenotypic identification methods, as the two species are closely related. Very little is known about the infectivity and virulence of this new species. In this study, *S. agnetis* was significantly more phagocytosed than *S. simulans*. However, in the killing assay, the results for *S. agnetis* were similar to those for *S. simulans* and even *S. aureus*, as these bacteria were not killed by murine macrophages.

*S. aureus* is commonly regarded to have a special status among mastitis-causing staphylococcal species in terms of the severity of inflammation, virulence, persistence and transmission. Although *S. aureus* mastitis can be severe and sometimes even toxic, it typically, and possibly after a clinical phase, causes chronic subclinical mastitis associated with a moderate or periodically high milk SCC. On the contrary, CNS rarely cause clinical mastitis, and the milk SCC in CNS mastitis usually varies from low to moderate, although the SCC may occasionally be high [5]. The mechanisms underlying the differences in the clinical manifestation of *S. aureus* and CNS mastitis are still unknown. In this study, *S. aureus* did not especially differ from the other *Staphylococcus* species regarding their phagocytosis or killing by macrophages. On the contrary, *S. simulans* differed most from the other *Staphylococcus* species in the phagocytosis assay and *S. chromogenes* in the killing assay.

Isolate differences in phagocytosis were seen in *S. simulans* and in killing for all the studied species. These results are partly the opposite to the study of Aarestrup et al. [20], who found significant differences between *S. aureus* strains in both phagocytosis and killing. However, Aarestrup et al. [20] used neutrophils, which migrate to the udder after the macrophages have initiated an inflammatory response.

In this study, none of the observed differences correlated with the nature of infection (clinical/subclinical). This may, however, be due to the small data sub-sets. Staphylococci can resist phagocytosis and killing in several ways. For example, the capsule and other extra-cellular polysaccharides are known to influence the efficacy of both phagocytosis and killing [31,38,39]. The *Staphylococcus* isolates used in this study all have a capsule/extra-cellular polysaccharide layer that can be visualized with Giemsa or Anthony’s capsule stain when grown in study conditions (Åvall-Jääskeläinen et al., unpublished observations). However, differences in these structures may exist that affect phagocytosis and/or killing. Other thus far unknown virulence factors can also have an impact on the observed resistance to phagocytosis and/or killing. Further studies are needed to examine these issues.

## Conclusions

*S. simulans* was significantly less phagocytosed by mouse macrophages than *S. agnetis* or *S. chromogenes*. In murine macrophages, *S. chromogenes* was more efficiently killed than any of the other *Staphylococcus* species studied. However, differences exist between isolates in their resistance to phagocytosis and killing by macrophages. Investigations at the pattern recognition level are needed to identify the mechanisms underlying these differences.

## Abbreviations

CFU: Colony forming unit; CNS: Coagulase-negative staphylococci; GM: Growth medium; PBS: Phosphate-buffered saline; PMN: Polymorphonuclear neutrophil; SCC: Somatic cell count; TLR: Toll like receptor.

## Competing interests

The authors declare that they have no competing interests.

## Authors’ contributions

ST, SÅJ and JK conceived and designed the experiments; SÅJ and JK conducted the laboratory experiments; SÅJ, JK and ST performed the data analysis and ST the statistical analysis; SÅJ, JK, ST and HS wrote the manuscript. All authors read and approved the final manuscript.
